# Microsatellite markers from tea green leafhopper *Empoasca* (*Matsumurasca*) *onukii*: a powerful tool for studying genetic structure in tea plantations

**DOI:** 10.1186/s12863-016-0420-3

**Published:** 2016-07-29

**Authors:** Li Zhang, Christopher H. Dietrich, Daozheng Qin

**Affiliations:** 1 Key Laboratory of Plant Protection Resources and Pest Management of the Ministry of Education; Entomological Museum, Northwest A&F University, Yangling, Shaanxi China; 2 Illinois Natural History Survey, Prairie Research Institute, University of Illinois, Champaign, IL USA; 3 Northwest A&F University, No.3 Taicheng Road, Yangling, Shaanxi 712100 China

**Keywords:** *Empoasca* (*Matsumurasca*) *onukii*, Microsatellite markers, Genetic differentiation

## Abstract

**Background:**

Tea green leafhopper is one of the most dominant pests in Chinese tea plantations. Recent evidence, including morphological and molecular data, revealed that tea green leafhopper in China is the same species as in Japan, *Empoasca* (*Matsumurasca*) *onukii* Matsuda. Previous morphological study that revealed variation in the structure of the male genitalia within and among populations of this species suggested that there may be significant population-level genetic variation. To provide powerful molecular markers to explore the population genetic diversity and population genetic structure of this pest in China, microsatellite markers were obtained by AFLP of sequences containing repeats (FIASCO).

**Results:**

Eighteen polymorphic markers were evaluated for five populations of *E.* (*M.*) *onukii*, Two related empoascine leafhopper species were selected to test the transferability of the markers. Population genetic structure of *E.* (*M.*) *onukii* was detected using Structure analysis, principal coordinate analysis (PCoA) and variance analysis. The identified markers were polymorphic with total number of alleles ranging from 6 to 24 per locus, observed and expected heterozygosity ranged from 0.133 to 0.9 and 0.183 to 0.926, respectively, and the polymorphic information content value over all populations varied from 0.429 to 0.911.

**Conclusions:**

This is the first study to demonstrate that microsatellite markers provide valuable information for genetic structure of *E*. (*M*.) *onukii* in Chinese tea plantations. There is obvious genetic differentiation between the two populations in the Southwest tea area. These microsatellite markers will be the powerful tools for genetic studies of *E*. (*M.*) *onukii* and improve understanding of the microevolution of this species.

**Electronic supplementary material:**

The online version of this article (doi:10.1186/s12863-016-0420-3) contains supplementary material, which is available to authorized users.

## Background

Tea green leafhopper, one of the most dominant pests in Chinese tea plantations recognized since the 1950s, causes considerable yield loss each year by piercing and sucking young leaves of tea [1, 2]. An incorrect scientific name, *Empoasca vitis* (Goëthe), was applied to this species for nearly 30 years in China and caused confusion in academic and applied research on the pest [3–6], but recent study of morphological and molecular evidence both revealed that tea green leafhopper in China is the same species as that occurring in Japan, *Empoasca* (*Matsumurasca*) *onukii* Matsuda [7, 8].

In China, tea-growing regions are fairly widespread across different climatic zones and tea green leafhopper has adapted to different habitats, conditions that may have given rise to genetic differentiation among populations. Previous studies have attempted to explore the genetic diversity of tea green leafhopper using either RAPD (Random Amplified Polymorphic DNA) techniques to assess the genetic polymorphism and relationships among seven populations of this species [9] or sequencing of mtDNA from the *COI* and 16S rRNA gene regions from different populations [10–12]. However, these studies were conducted without first confirming the species identifications of the leafhoppers included, using morphology. Because of the high species diversity (>800 species worldwide and >200 in China) and morphological similarity of the Chinese fauna of *Empoasca* it is important to examine the male genitalia of specimens used in such studies to ensure accurate identification. Also in these previous studies only a few genetic markers were used for a single individual, and their variation was too low for studying population structure. As a result, the population structure of *E.* (*M.*) *onukii* is still poorly understood.

Based on their high polymorphism, codominance, abundance, and stability, microsatellite markers have been widely adopted to obtain multilocus genotypes using minute quantities of DNA [13, 14]. They are also known to provide more information than a single marker (such as mtDNA) on population genetic differentiation, allowing study of colonization patterns and population dynamics using simple statistical procedures [15]. Only three previous studies of leafhopper pest populations have incorporated microsatellite data. Papura et al. isolated ten and eight microsatellite markers, respectively, for *Scaphoideus titanus* Ball and *Empoasca vitis* (Goëthe), and subsequently showed that European *S. titanus* populations originated from northeastern North America [16–18]. Shabani et al. suggested that climatic and/or geogeraphical barriers might induce population genetic differentiation of the leafhopper *Hishimous phycitis* using the mitochondrial cytochrome oxidase I (*COI*) gene and nine microsatellite DNA markers isolated by FIASCO [19, 20]. Unfortunately, two of the three leafhopper species included in these previous studies are distantly related to *Empoasca*, belonging to a separate cicadellid subfamily (Deltocephalinae), and we were unable to consistently amplify the microsatellite markers developed for these species in Chinese *E.* (*M.*) *onukii* populations.

Tea production originated in Southeast China more than 3000 years ago and is now widespread in tropical and subtropical regions of the world [21]. Our previous comparative morphological study revealed variation in male genitalia among individuals taken from different populations of *E.* (*M.*) *onukii* [7, 22]. This suggests that there may be broader underlying genetic differences that could be revealed through study of molecular data. According to the previously recognized divisions of Chinese tea production areas, the provinces of Henan, Shandong and Shaanxi belong to the Jiangbei tea area and have different climate and topography from the provinces of Sichuan and Yunnan in the Southwest tea area [23]. Given the substantial climatic differences between the Jiangbei and Southwest tea areas, as well as the existence of geographic barriers (e.g., mountain ranges, large rivers) that may restrict gene flow both within and between these areas, genetic differentiation might be expected to occur among *E.* (*M.*) *onukii* populations both within and between the recognized areas.

The aim of this study was to develop microsatellite markers and to use them to analyze genetic structure of *E.* (*M.*) *onukii* populations in the Jiangbei and Southwest tea areas. These microsatellite markers will provide the tools needed for genetic studies of *E*. (*M.*) *onukii* aimed at elucidating the microevolution and population dynamics of this species in China. This information will help pinpoint the origin of this pest and its routes of dispersal, which will be used to develop environmental friendly control strategies against this species in different tea areas.

## Methods

### Samples collections

**Fig. 1:**
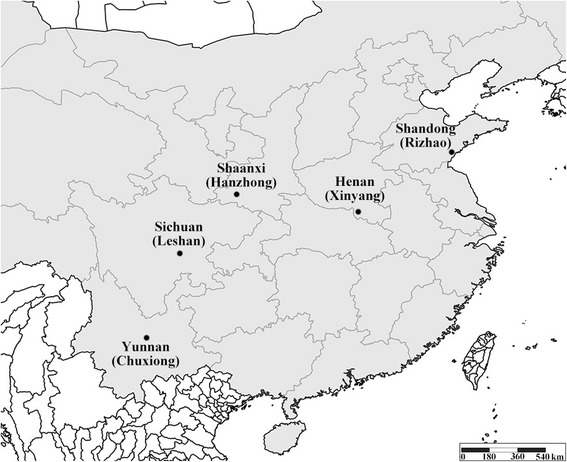
Geographical distribution of specimen sites of *E.* (*M.*) *onukii* in different locations. City names are in parentheses after province names that are the specimen population ID’s; *dots mark* locations of collection sites

Tea green leafhopper specimens used in this study were collected from tea plantations in five provinces representing five geographic populations (see Fig. 1 and Table 1). To reduce the likelihood of sample contamination by non-target species, specimens were collected by sweep net from the middle of tea plantings in areas without weeds and non-tea plants. Specimens representing each population were carefully collected from five sites near the central areas in three tea plantations. At least 50 male individuals were collected in each province and thereafter identified by the corresponding author in the laboratory using morphological characters described previously [7]. All the specimens are now deposited in the Entomological Museum, Northwest A&F University, Yangling, China (NWAFU) in absolute alcohol at −20 °C.

**Table 1:** Collecting information of five included *E.* (*M.*) *onukii* populations

Province (City)	Longitude(E)/Latitude(N)	Collecting date (M/Y)	Population ID	No. of male individuals
			Province (tea area)	
Henan (Xinyang)	31°45.90′/114°40.03′	7/2013	Henan (Jiangbei)	230
Shandong (Rizhao)	35°17.02′/119°16.00′	7/2013	Shandong (Jiangbei)	124
Shaanxi (Hanzhong)	32°57.00′/107°40.11′	7/2013	Shaanxi (Jiangbei)	189
Sichuan (Leshan)	29°46.50′/103°40.50′	9/2013	Sichuan (Southwest)	69
Yunnan (Chuxiong)	24°32.62′/101°49.78′	7/2014	Yunnan (Southwest)	107

### Screening microsatellite from enriched libraries

Microsatellites were enriched and isolated by FIASCO [24, 25]. Genomic DNA was extracted from a pool of 10 specimens (with genital segments removed for morphological identification) by a modified CTAB protocol [26]. DNA concentration was measured using an ND-1000 spectrophotometer (Bio-Rad, Hercules, CA, USA). The (AG)_12_, (AC)_12_ and (AAC)_8_-enriched partial genomic libraries were constructed by FIASCO. 100 ng of DNA was digested with *Mse*I (BioLabs, Beijing, China) and ligated to prepared *Mse*I AFLP adaptors (5′-TACTCAGGACTC AT-3′/5′-GACGATGAGTCCTGAG-3′) using T4 DNA ligase (TaKaRa, Dalian, China) The linker-adaptor-PCR was performed in a final volume of 50 μL containing: 2 mM MgCl_2_, 250 uM dNTP, 0.8 uM *Mse*I AFLP adaptors and 0.32 U of Taq DNA polymerase (TaKaRa, Dalian, China). The reaction procedure included denaturing at 94 °C for 3 min, followed by 20 cycles of 30 s at 94 °C, 1 min at 53 °C, 1 min at 72 °C, and a final extension of 10 min at 72 °C. 300–750 bp DNA fragments were purified and separated using a QIAquick PCR Purification Kit (QIAGEN, Shanghai, China). The product was then denatured for 5 min at 95 °C and hybridized with biotinylated probes ((AC)_12_, (AG)_12_ and (AAC)_8_) for 2–3 h, respectively.

DNA fragments were selectively captured by streptavidin-coated magnetic breads (10 mg/μL, Dynalbeads M-280 Streptavidin, Invitrogen). Nonspecific binding and redundant DNA was eluted by several non-stringent and stringent washes. The microsatellite-enriched DNA fragments were amplified with *Mse*I-N (5′-GATGAGTCCTGAGTAAN-3′). The PCR products were ligated to pMD19-T vectors (TaKaRa, Dalian, China) and transformed into *Escheri-chia. coli* strain *Trans*1-T1 as follows: cells were cultured at 37 °C for about 16 h on LB agar plates containing ampicillin, X-gal, and IPTG for blue/white selection. Insert-positive bacterial clones were transfered into liquid medium with ampicillin in 96-well plates and cultivated at 37 °C for about 4 h. PCR amplification was performed in a volume of 25 μL containing: 1 μL bacterium solution, 2 mM MgCl_2_, 250 uM dNTP, 0.8 uM each of M13 forward and M13 reverse primer and 0.32 U of Taq DNA polymerase (TaKaRa, Dalian, China). The reaction procedure included denaturing at 94 °C for 3 min, followed by 35 cycles of 30 s at 94 °C, 40 s at 57 °C, 50 s at 72 °C, and a final extension of 10 min at 72 °C. PCR products were considered as positive clones when two or more bands appeared in the 100–500 bp size range and were sequenced by Sangon (Shanghai, China) after purification.

### Primer design and preliminary evaluation of amplification

The results were screened for SSR motifs by SSR Hunter 1.01 [27]. Primers were designed based on sequences containing four or more microsatellite repeats using Primer 5.0 [28]. Following primer synthesis, DNA of *E.* (*M.*) *onukii* individuals was amplified in 10 μL total volume with 20 ng DNA and containing 2.5 mM MgCl_2_, 250 uM dNTP, 1uM each of forward and reverse primer and 0.2 U of Taq DNA polymerase (TaKaRa, Dalian, China).

All of the primers were screened by “touchdown PCR”, which included 4 min denaturation at 94 °C: 30 s at 94 °C, 30 s at 50 °C to 60 °C (dropping 0.3 °C/cycle), 30 s at 72 °C, 30 cycles, then 35 cycles of 30 s at 94 °C, 30 s at 57 °C, 30 s at 72 °C, and one cycle of 7 min at 72 °C. Thereafter, the optimum annealing temperature of primers was determined by gradient temperature PCR, including a 4 min denaturation at 94 °C, 30 s at 94 °C, 30 s at 50 to 65 °C (dropping 1 °C/sample), 30s at 72 °C, 30 cycles, then 7 min at 72 °C. Finally, fragment length polymorphism of products amplified with the optimum annealing temperature were further analyzed by 6 % polyacrylamide gel electrophoresis for samples representing eight individuals from four *E.* (*M.*) *onukii* populations. From the results of electrophoresis, polymorphism was defined as the presence of more than two alleles (bands of different size). Polymorphic markers were selected based on their performance in PCR and number of alleles.

### Polymorphism evaluation in *E.* (*M.*) *onukii* populations and cross-species amplification

Thirty-one selected primers were synthesized and labeled as forward primers (FAM). To obtain accurate allele frequencies, 30 individuals per population (150 individuals in total) collected from the five different populations were used to evaluate polymorphism (Table 1). All primers were tested for cross-amplification in the related non-target species *Empoasca* (s. str.) sp. (*n* = 8) and *Alebrasca actinidiae* Hayashi & Okada (*n* = 8). Genomic DNA was extracted from each single individual by CTAB, yielding a concentration of > 20 ng/μl and polymorphism was assessed by using labeled primers and the PCR protocol noted above. PCR products were then run by automated capillary electrophoresis using a genetic analyzer (3130xl; ABI, Foster, CA, USA). Data were analyzed using GeneMapper v4.0 (Applied Biosystems, Foster City, CA, USA).

### Genetic diversity analysis

The frequency of null alleles was evaluated by Micro-Checker [29]. The number of alleles per polymorphic marker (*A*), observed heterozygosities (*H*_O_), expected heterozygosities (*H*_E_) and polymorphism information content (PIC) were calculated by Cervus 2.0 [30]. The allelic richness (*AR*) was estimated using a minimum sample size of 25 diploid individuals in HP-Rare v1.0 [31]. Deviations from Hardy-Weinberg equilibrium (HWE) across markers in different populations were calculated by Genepop v3.4 [32]. Linkage disequilibrium (LD) between pairs of markers was carried out with Genepop v3.4. P values were corrected for multiple tests by applying the sequential Bonferroni correction [33]. Independent samples *t*-test in Spss Statistics 20 (IBM) was used to examine if the genetic diversity significantly differed between the Jiangbei and Southwest populations. Differences in allelic frequencies were tested with Fisher’s method (exact G test) using Genepop v3.4.

### Genetic structure analysis

Population structure was defined on the basis of Structure analysis and principal coordinate analysis. Structure 2.3.4 was used to generate clusters of individual genotypes by Bayesian assignment [34]. An admixture ancestry model and the correlated allele frequency model were used to calculate the log likelihood of the data (L(K)) [34]. 20 independent runs for each K (K = 1–10) were carried out with a burn-in period of 50,000 iterations in 1,000,000 Markov Chain Monte Carlo (MCMC) repetitions. The number of genetic clusters (K) among the five populations was determined by the log likelihood of the data (L(K)) and the ad hoc statistic (ΔK) estimated the second order rate of change in L(K) between successive K [34–36]. The principal coordinate analysis (PCoA) based on Phi-st distances (GD) was performed by GenALEX 6.502 [37]. Nei’s genetic distance was obtained by Popgene v1.32 [38, 39].

Analyses of molecular variance (AMOVA) and fixation indices were performed by Arlequin 3.11 [40]. AMOVA analysis detected genetic differentiation at three hierarchical levels: 1) among groups (i.e. group 1 included the Shandong, Henan and Shaanxi populations; the Sichuan and Yunnan populations belonged to groups 2 and 3, respectively); 2) among populations within groups (i.e. among the Shandong, Henan and Shaanxi populations); and 3) within populations (i.e., among individual leafhoppers in the same population). The significance of the inter-individual and inter-population variance components were tested with 10,000 permutations. Fixation indices and their significance were tested with 1000 permutations. Arlequin 3.11 was also used to compute the degree of genetic differentiation among five populations as measured by population specific *F*_ST_ indices.

## Results

### Evaluation of microsatellite markers

One hundred and seventy-three clones were selected to be sequenced from three microsatellite-enriched libraries, (AC)n, (AG)n and (AAC)n. After searching for repeats, 74 sequences were deemed adequate for designing primers. 56 pairs of primers were obtained, 31 of which successfully yielded clear single target bands of predicted size, with the others showing multi-banding patterns or no amplification when products were visualized on 2 % agarose gels. 21 markers showed fragment length polymorphism, accounting for 37.5 % of total markers. The sequences of the 21 markers have been uploaded to GenBank and accession numbers are shown in Additional file 1: Table S1. 18 labeled primers were also polymorphic, based on polymorphism evaluation of automated capillary electrophoresis across five *E.* (*M.*) *onukii* populations (Additional file 2: Table S2). Eight of these were successfully amplified in the related species *Empoasca* (s. str.) sp. (collected from Anhui, China) and three in the more distantly related *Alebrasca actinidiae* Hayashi & Okada (collected from Hunan, China) (Table 2). Five of eight markers were polymorphic in *E*. (s. str.) sp. However, for *A. actinidiae*, all three amplified markers yielded single amplified fragments.

**Table 2:** Transferability of eight microsatellite markers from *E.* (*M.*) *onukii* to related species *Empoasca* (s. str.) sp. and *Alebrasca actinidiae*

Species	*Empoasca* (s.str.) sp.				*Alebrasca actinidiae* Hayashi & Okada			
Marker	Size range (bp)	*A*	*H*_O_	*H*_E_	Size range (bp)	*A*	*H*_O_	*H*_E_
*Eo*-51	242	1	-	-	-	-	-	-
*Eo*-37	163–169	4	0.400	0.711	-	-	-	-
*Eo*-1-61	90–102	3	0.143	0.385	-	-	-	-
*Eo*-42	109–123	4	0.500	0.643	123	1	-	-
*Eo*-E-12	159–163	3	0.000	0.667	159	1	-	-
*Eo*-4--5	203–213	1	-	-	213	1	-	-
*Eo*-70	222	1	-	-	-	-	-	-
*Eo*-1--5	119–131	2	0.143	0.143	-	-	-	-

*A* number of alleles, *H*_E_ expected heterozygosity, *H*_O_ observed heterozygosity, −, amplication failed (no, faint or multiple band)

### Genetic diversity

*Eo*-4-5, *Eo*-1-77 and *Eo*-F-8 exhibited a significant excess of homozygosity and the amplication rates were lower than 50 %. So rejecting *Eo*-4-5, *Eo*-1-77 and *Eo*-F-8, the remaining 18 markers were selected for population genetic studies. After Bonferroni correction, nine markers deviated from Hardy-Weinberg equilibrium (pHWE < 0.01) in different populations, but linkage disequilibrium was not detected for any pair of markers. *Eo*-54, *Eo*-1-61, *Eo*-1-52, *Eo*-83, *Eo*-E-12, *Eo*-70, *Eo*-36, *Eo*-1-65 and *Eo*-1-5 deviated from HWE because of null alleles (Table 3), but null allele frequencies were low. The total number of alleles for the 18 markers varied from 6 to 24. The polymorphic information content (PIC) value over all populations varied from 0.429 to 0.911 (Table 3). Except for *Eo*-1-5, 17 markers had high polymorphism, with PIC value above 0.5 [41].

**Table 3:** The genetic diversity of 18 microsatellite markers in five *E*. (*M*.) *onukii* populations

Microsatellite markers	*A*	*H*_O_	*H*_E_	PIC	Frequency of null allele
*Eo*-29	10	0.757	0.697	0.660	−0.048
*Eo*-51	18	0.755	0.862	0.844	0.055
*Eo*-37	8	0.616	0.652	0.611	−0.009
*Eo*-54	19	0.760	0.907	0.896	0.061
*Eo*-1-61	13	0.581	0.717	0.691	0.044
*Eo*-1-52	13	0.537	0.799	0.773	0.015
*Eo*-42	11	0.396	0.444	0.429	0.043
*Eo*-1-82	15	0.786	0.859	0.842	0.004
*Eo*-9	13	0.562	0.813	0.792	0.121
*Eo*-20	17	0.767	0.909	0.898	0.066
*Eo*-1-57	6	0.453	0.654	0.583	0.053
*Eo*-83	11	0.378	0.574	0.549	0.152
*Eo*-E-12	22	0.557	0.919	0.911	0.132
*Eo*-70	24	0.518	0.912	0.903	0.142
*Eo*-36	16	0.519	0.867	0.849	0.110
*Eo*-68	16	0.557	0.784	0.752	0.115
*Eo*-1-65	13	0.433	0.725	0.676	0.084
*Eo*-1-5	10	0.376	0.561	0.541	0.148

PIC, *H*_E_, *H*_O_ and *A* refer to as the total polymorphic information content value and expected heterozygosity, observed heterozygosity and number of alleles per locus over all populations

The number of alleles for the 18 markers varied from 4 to 18 among different populations. The mean number of alleles and allelic richness per population ranged from 8.7 to 9.4 and from 8.4 to 9.0, respectively. The mean expected and observed heterozygosity per population ranged from 0.665 to 0.758 and from 0.516 to 0.632, respectively (Additional file 2: Table S2). The mean allelic richness was significantly different between the Jiangbei and Southwest populations (*t*-test: t = 5.400, d.f. = 3. *P* = 0.012). Similarly, the mean expected heterozygosity were significantly different between the Jiangbei and Southwest populations (*t*-test: t = 3.763, d.f. = 3. *P* = 0.033). Analysis of allelic frequencies across all markers showed significant differences between each population pair (Fisher’s method, G test, *P* < 0.05). Allelic frequencies for each marker in each population were shown in Additional file 3: Table S3.

### Genetic structure

**Fig. 2:**
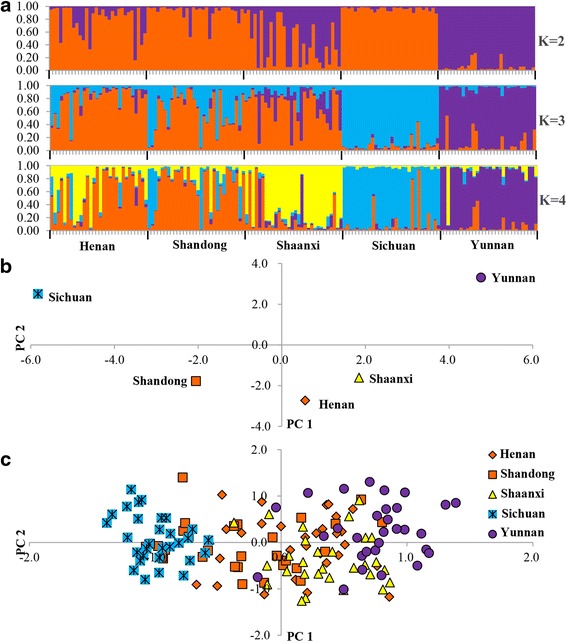
Genetic structure of tea green leafhopper populations by Structure analysis and PCoA. **a** Barplots of Structure analysis for K = 2–4. Each bar represents one individual leafhopper and each color represents by a cluster. The number of clusters inferred was K = 4. The Henan, Shandong and Shaanxi populations belong to the Jiangbei populations. Sichuan and Yunnan populations belong to Southwest populations. **b** PCoA at population level. Each label represents one population. **c** PCoA by individuals Each leafhopper is represented by the label corresponding to its population origin. **b** and **c** The different colors represent the major cluster inferred by Structure analysis

Results of Bayesian Structure analysis are shown in Fig. 2a. Although ΔK had peaks in K = 2 and K = 4, the estimated value of L(K) was higher for K = 4 than for K = 2 (Additional file 4: Figure S1). The numbers of individuals from Henan assigned to each cluster are similar to those of the Shandong population. The individuals from Shaanxi were assigned to a cluster different from individuals in the Henan and Shandong populations at K = 4. Thus, the most likely value of K was 4, suggesting division into four genetically distinct groups. Whether the value of K was 2, 3 and 4, the Sichuan and Yunnan populations were largely separated into two clusters, indicating that these two geographic populations were clearly genetically differentiated. Similar results were obtained using PCoA for individuals and populations, showing in Fig. 2. For populations, percentages of variation explained by principal coordinate 1 (PC 1) and principal coordinate 2 (PC 2) were 59.65 % and 28.21 %, respectively, in Fig. 2b, showing clearly separated these populations. The Sichuan population was distant from the Yunnan population. The Shaanxi population was close to the Henan and Shandong populations. For individuals, the cumulative percentages of first two eigen values was 15.55 % in Fig. 2c, showing a clear differentiation between the Sichuan and Yunnan populations and only partially distinguished the Henan, Shandong and Shaanxi populations.

The pairwise Nei’s genetic distances among the Henan, Shandong and Shaanxi populations were 0.0838–0.1466 (Table 4). The pairwise genetic distances between the Jiangbei and Southwest populations were 0.1414–0.3886. The lowest genetic distance is 0.0838, between the Henan and Shandong populations. The highest genetic distance is 0.3886, between the Sichuan and Yunnan populations.

**Table 4:** Pairwise *F*_ST_ (above diagonal) and genetic distance (below diagonal) of five populations

Pop ID	Henan	Shandong	Shaanxi	Sichuan	Yunnan
Population (tea area)	(Jiangbei)	(Jiangbei)	(Jiangbei)	(Southwest)	(Southwest)
Henan (Jiangbei)	****	0.0158	0.0206	0.0901	0.0408
Shandong (Jiangbei)	0.0838	****	0.0415	0.0486	0.0636
Shaanxi (Jiangbei)	0.0847	0.1466	****	0.1056	0.0376
Sichuan (Southwest)	0.2525	0.1414	0.2734	****	0.1277
Yunnan (Southwest)	0.2124	0.2791	0.1757	0.3886	****

All *F*_ST_ have *P* < 0.05. **** separated *F*_ST_ and genetic distances

Analysis of molecular variance (AMOVA) revealed that majority of the genetic variance originated from variation among individuals within populations (92.78 %) and was highly significant (FST = 0.07219, *P* < 0.0001). The proportion of genetic variance was larger between groups (4.56 %) than between populations (2.66 %). The fixation indices between groups (FCT = 0.04560, *P* < 0.0001) and between populations (FSC = 0.02787, *P* < 0.0001) were significant (Additional file 5: Table S4). The *F*_ST_, among the Henan, Shandong and Shaanxi populations, ranged from 0.0158 to 0.0415, revealing low genetic differentiation. There was higher difference in the *F*_ST_ between the Sichuan and other populations, ranging from 0.0901 to 0.1277. The *F*_ST_ between the Yunnan and other populations, ranged from 0.0376 to 0.1277 (Table 4), representing moderate differentiation. The largest difference in the *F*_ST_ was 0.1277, between the Sichuan and Yunnan populations.

## Discussion

Although previous studies reported 27 microsatellite markers for three different leafhopper species [16, 17, 20], our attempts to amplify these markers in *E.* (*M.*) *onukii* populations failed. This is not surprising, given that two of the three species in these previous studies belong to the distantly related leafhopper subfamily Deltocephalinae and that leafhoppers, in general, are a phyletically diverse and widespread group of insects. The results of cross-species amplification indicate that, in this group of leafhoppers, microsatellite markers are highly species-specific, and amplification rate of markers developed for one species decrease proportionally according to the genetic distance in other species [13].

Sufficient numbers of specimens from each isolated population and tests of polymorphism for markers are the key factors for successfully developing microsatellite markers. Accordingly, tea leafhopper specimens in this study were collected from sites at least 500 km distant from each other. Following the recommendations of Hale et al. [42], we used 30 individual leafhoppers from each site to screen 21 markers using the following criteria: ease of amplification; detection of fragment length polymorphism by polyacrylamide gel electrophoresis; evaluation of population-level polymorphism. The polymorphism of the developed microsatellite markers is moderate to high and shows uneven distribution of alleles, consistent with microsatellite markers of other leafhoppers [16, 17]. Failure of markers *Eo*-4-5, *Eo*-F-8,*Eo*-1-77 due to lack of amplification, monomorphic fragments or unstable polymorphism, may have various causes including mutations within primer regions or presence of secondary DNA structures that prevented amplification. Among the 18 remaining markers, *Eo*-54, *Eo*-1-61, *Eo*-1-52, *Eo*-83, *Eo*-E-12, *Eo*-70, *Eo*-36, *Eo*-1-65 and *Eo*-1-5 deviated from HWE because of null alleles and a deficit of heterozygotes. But null allele frequency of 18 markers, ranged from −0.048 to 0.152, below 0.200 and induced little effect on the result of genetic diversity and genetic structure [43, 44]. As a result, 18 microsatellite markers can be used in the further study of genetic structure in Chinese *E.* (*M.*) *onukii* populations.

High genetic diversity among *E.* (*M.*) *onukii* populations revealed by 18 microsatellite markers is probably due to some combination of geographic isolation and climatic variation among the tea-growing regions in which these populations occur. Genetic diversity and genetic structure based on 18 microsatellite markers highlighted the existence of significant genetic difference between the Sichuan and Yunnan populations in the topographically complex Southwest tea area. Furthermore, different mean allelic richness and mean expected heterozygosity were found between the Jiangbei and Southwest populations (Additional file 2: Table S2). The Structure analysis also revealed different genetic diversity between the two areas (Fig. 2a). Yunnan population also showed distinct genetic differentiation, consistent with analysis of mitochondrial gene variation [10]. There is moderate level of differentiation between the Sichuan and the other populations. The highest level of differentiation appeared between the Sichuan and Yunnan populations (Table 4), and this result was consistent with the result of PCoA for populations and Structure analysis. Tea green leafhoppers appear to have limited dispersal capacity and live in a relatively isolated mountainous and basin environment in the Southwest tea area, which may explain the high genetic differentiation among populations [10, 12]. At K = 4, the Shaanxi population was separated from the Henan and Shandong populations (Fig. 2a). This may be attributed to the barrier formed by the Qinling Mountains between the Shaanxi and other populations of the Jiangbei tea area. A significant amount of the diversity was shared between individuals in different populations, especially for Henan, Shandong and Shaanxi populations, based on Structure analysis and PCoA for individuals. AMOVA indicated that the genetic variation mainly derived from individual variation, and the fixation indices among groups is larger than among populations within groups. These results suggest high gene flow and low genetic differentiation among the Henan, Shandong and Shaanxi populations, perhaps due to the anthropogenic transport and similar climatic conditions.

The microsatellite markers developed here revealed genetic differentiation of *E.* (*M.*) *onukii* in the Jiangbei and Southwest tea areas. However, the data available so far are not sufficient to reconstruct invasion routes. The markers developed here will be used in future, more detailed, analyses of genetic structure of *E.* (*M.*) *onukii* populations in Chinese tea plantations and to study the evolutionary mechanisms yielding the observed variation.

## Conclusion

Seventy-four *E*. (*M*.) *onukii* microsatellite sequences were obtained and analyzed. 18 polymorphic markers were selected to analyze five populations of *E*. (*M*.) *onukii*. Study of the genetic structure of five Chinese populations demonstrate that the newly developed markers provide valuable information on the genetic structure of *E*. (*M*.) *onukii* in Chinese tea plantations. The Structure analysis and PCoA for populations reveals that there is significant genetic differentiation between the Sichuan and Yunnan populations and that these have similar genetic diversity to that present among the Henan, Shandong and Shaanxi populations. These microsatellite markers will be powerful tools for genetic study of *E*. (*M*.) *onukii* and yield an improved understanding of the microevolution of this species.

## Additional files

Additional file 1: Table S1. Characteristics of 21 microsatellite markers in *E*. (*M*.) *onukii*. (XLS 27 kb) Additional file 2: Table S2. Number of alleles and heterozygosity in each *E*. (*M*.) *onukii*. population. (XLS 29 kb) Additional file 3: Table S3. Allelic frequencies of 21 microsatellite markers from Genepop for five populations. (XLS 56 kb) Additional file 4: Figure S1. Estimated number of genetic clusters obtained with Structure for K value ranging from 1 to 10 using 18 microsatellite markers for all populations. a graph of estimated mean log likelihood (L(K)). b graph of ad hoc statistic (ΔK). The most likely value of K was 4. (PDF 568 kb) Additional file 5: Table S4. AMOVA result of five *E*. (*M*.) *onukii* populations among two groups. (XLS 20 kb)
