# Supplementary figures and images for: Microsatellite markers from tea green leafhopper Empoasca (Matsumurasca) onukii: a powerful tool for studying genetic structure in tea plantations
Source: BMC Genet. 2016 Jul 29;17:112. doi: 10.1186/s12863-016-0420-3 (PMC4966850; doi:10.1186/s12863-016-0420-3)

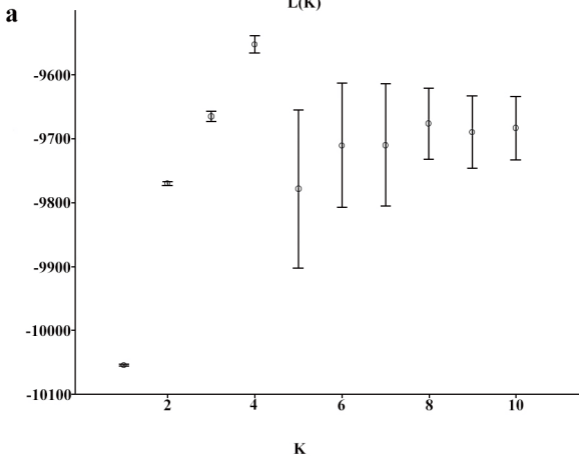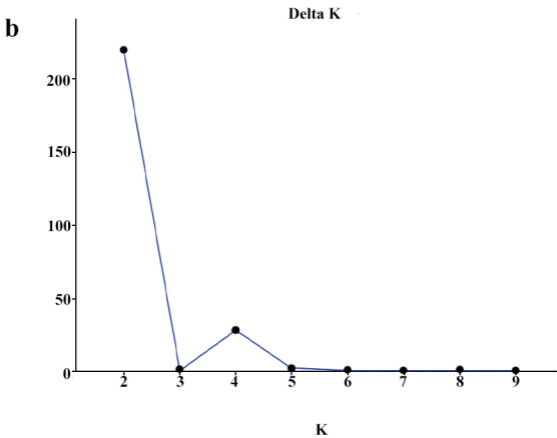

Supplement: Additional file 4: Figure S1. — Estimated number of genetic clusters obtained with Structure for K value ranging from 1 to 10 using 18 microsatellite markers for all populations. a graph of estimated mean log likelihood (L(K)). b graph of ad hoc statistic (ΔK). The most likely value of K was 4. (PDF 568 kb) [file 12863_2016_420_MOESM4_ESM.pdf]
